# Clinical Application of a Two-Step Percutaneous Nephrolithotomy in a Patient With Severe Kyphoscoliosis and a Malrotated Kidney

**DOI:** 10.7759/cureus.17340

**Published:** 2021-08-20

**Authors:** Mohammad A Alomar, Omar S Alghamdi, Mohammad A Alghafees, Razan A Alhamidi, Alhasan M Abduldaem, Mohammed M Aljohani

**Affiliations:** 1 Urology, King Faisal Specialist Hospital and Research Centre, Riyadh, SAU; 2 College of Medicine, King Saud University, Riyadh, SAU; 3 College of Medicine, King Saud Bin Abdulaziz University for Health Sciences, Riyadh, SAU

**Keywords:** percutaneous nephrolithotomy (pcnl), kyphoscoliosis, endo urology, renal calculi, spinal deformiites

## Abstract

This case report describes a two-step percutaneous nephrolithotomy (PCNL) in a 22-year-old male who had severe kyphoscoliosis and a malrotated kidney. The operation was performed with the patient under general anesthesia and in the left lateral decubitus position. All stones were successfully removed. No complications occurred during surgery, and the patient recovered well. Regardless of the posed challenges for kidney stone treatment in patients with spinal deformities, PCNL is not only a minimally invasive but also a safe and effective treatment option when done under correct positioning. The success rate is high, and the morbidity rate is low. According to the literature, only 125 cases of PCNL implications in kyphoscoliosis patients have been reported in emerging case reports and case series.

## Introduction

Kidney stones are a common condition. In the United States, nearly one in 11 people is affected at some stage in their lives [1]. The incidence is as high as 14.8% and is increasing [2]. These stones are also likely to reappear, and at least 50% of people experience another stone within 10 years of their first onset [2]. Hypertension, diabetes, obesity, and metabolic syndromes are considered risk factors for the formation of stones, which in turn could lead to chronic kidney disease and end-stage renal disease. In our study, a patient was diagnosed with kyphoscoliosis which involves lateral and rotational deformities of the spine and occurs in less than 4% of the population. Approximately, 70% of the cases are idiopathic. The remaining 30% are secondary to congenital diseases, neuromuscular diseases, mesenchymal diseases, infections, or traumas [3]. Patients with kyphoscoliosis may have secondary problems affecting the urinary tract, such as infections, stone formations, and kidney failure, which may impact their quality of life. Several treatments have been used to treat kidney stones, including shock wave lithotripsy (SWL), ureteroscopy, percutaneous nephrolithotomy (PCNL), and open surgery [4].

Anatomical entities can prevent the use of shock waves in a large number of these patients [5]. Therefore, more invasive methods, such as PCNL, may be needed to achieve adequate stone removal. PCNL is the gold standard in the treatment of kidney stones > 2 cm and achieves a success rate ranging between 74% and 100% [6]. However, performing percutaneous surgery may also be affected by abnormal anatomy. The synchronous presence of scoliosis with kidney stones significantly increases the surgical risk of PCNL due to anatomical deformation and thus constitutes a technical barrier to PCNL. The American Urological Association guidelines list severe scoliosis as a contraindication for PCNL [7]. This report describes the utilization of a PCNL in a patient with severe acquired kyphoscoliosis. During the surgery, a trocar was precisely implanted under type B ultrasound guidance and the kidney stone was successfully removed by a two-stage PCNL.

## Case presentation

**Figure 1:**
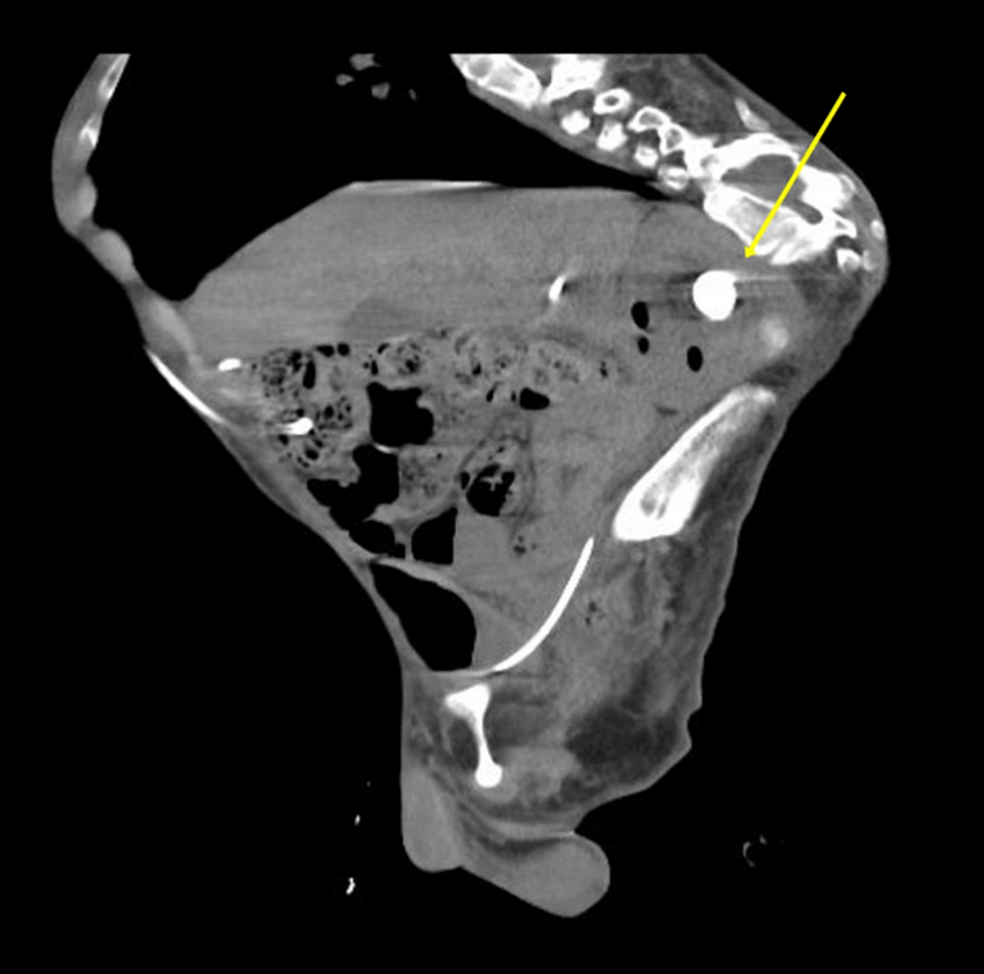
A sagittal view of the CT scan showing a radiopaque staghorn stone (yellow arrow) in a malrotated kidney with mild hydronephrosis. CT: computed tomography.

**Figure 2:**
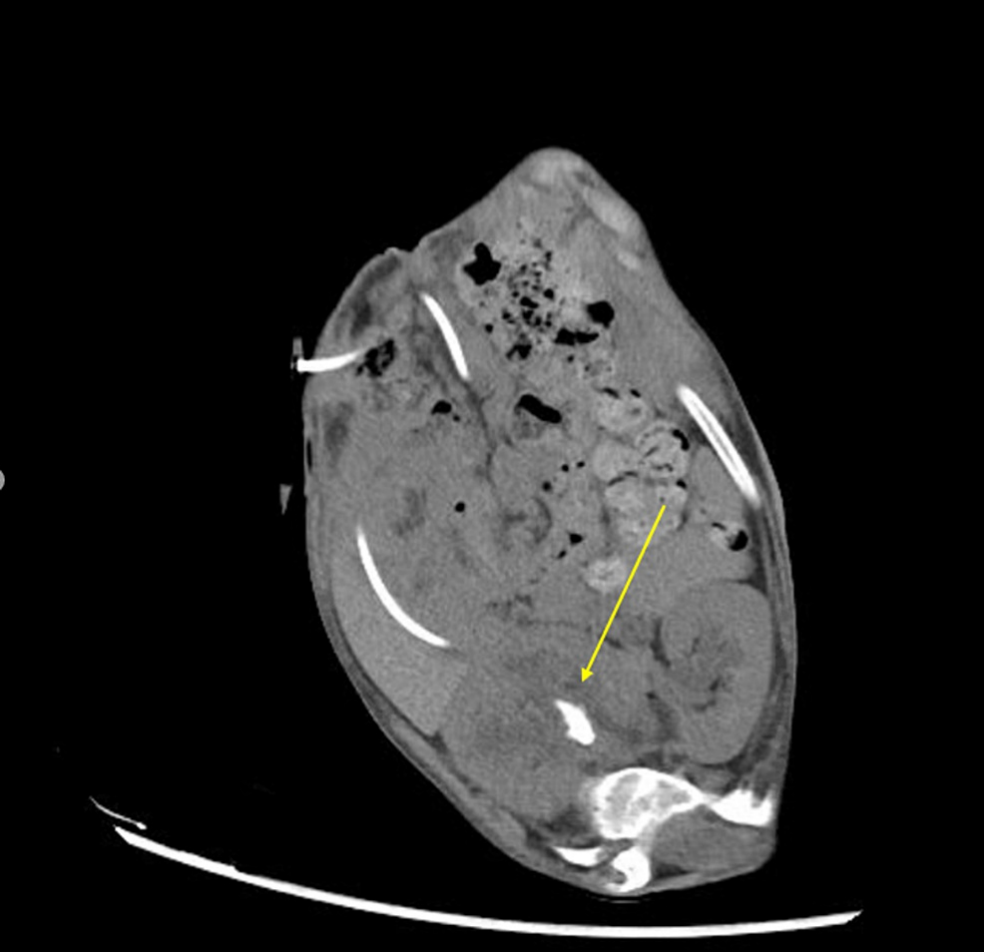
An axial view of the CT scan showing a radiopaque staghorn stone (yellow arrow) in a malrotated kidney with mild hydronephrosis. CT: computed tomography.

A 22-year-old male was referred to our setup with a right renal calculus. He has a known case of myelomeningocele, a neurogenic bladder, and severe kyphoscoliosis. His surgical history is significant for a bladder neck suspension, a mitrofanoff creation, a cecal tube insertion, a right inguinal herniotomy, a cystolitholapaxy, and a ventriculoperitoneal shunt insertion. He denied other associated symptoms like fever, flank pain, and vomiting. On examination he was well-looking, vitally stable, and afebrile. On exam, the abdomen was soft and lax. He also had a mitrofanoff and a cecostomy. Initial baseline investigations were unremarkable. A computed tomography (CT) scan without contrast was done and showed a right staghorn calculus measuring 2.1 cm which was associated with mild hydronephrosis (Figures 1, 2).

**Figure 3:**
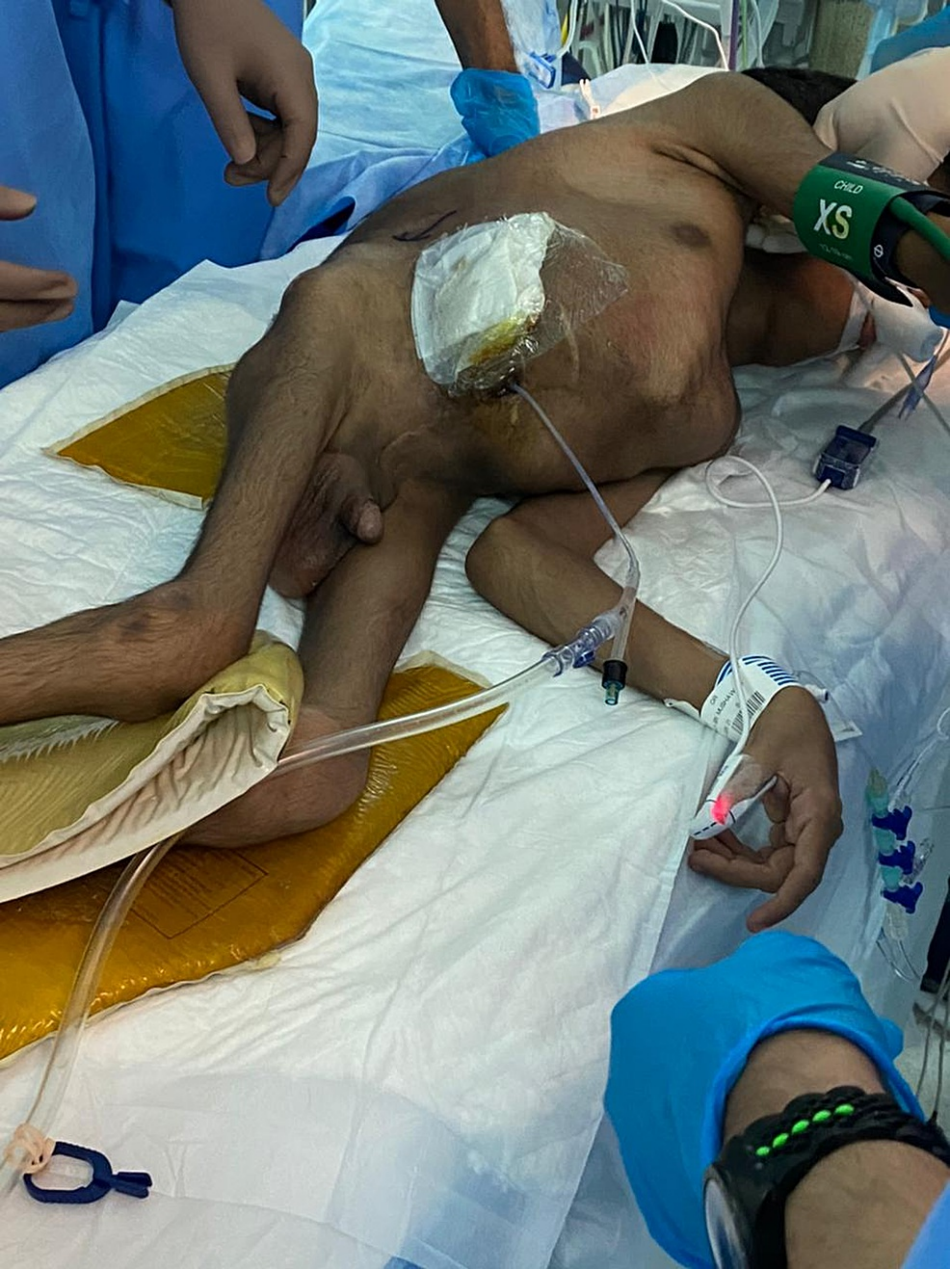
A preoperative image of the patient following his positioning at the left lateral decubitus position and the insertion of the catheter through the mitrofanoff.

A PCNL approach was discussed and performed. After induction of general anesthesia and insertion of the catheter through the mitrofanoff, the patient was positioned at the left lateral decubitus position with the right side up due to severe kyphosis of thoracolumbar vertebra (Figure 3).

**Figure 4:**
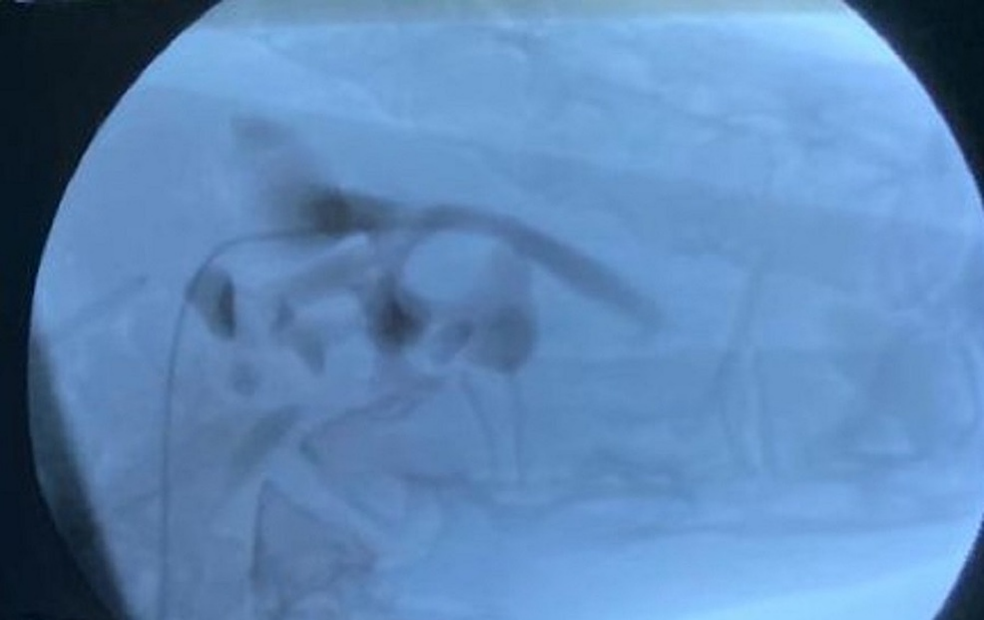
An intraoperative fluoroscopy image after inserting the guidewire and gaining access to the right kidney.

Prepping and draping were done in a sterile manner. An interventional radiologist was involved to access the kidney using ultrasound and fluoroscopic guidance. After the access, two guide wires were secured, one safety guidewire and one working guidewire (Figure 4)

**Figure 5:**
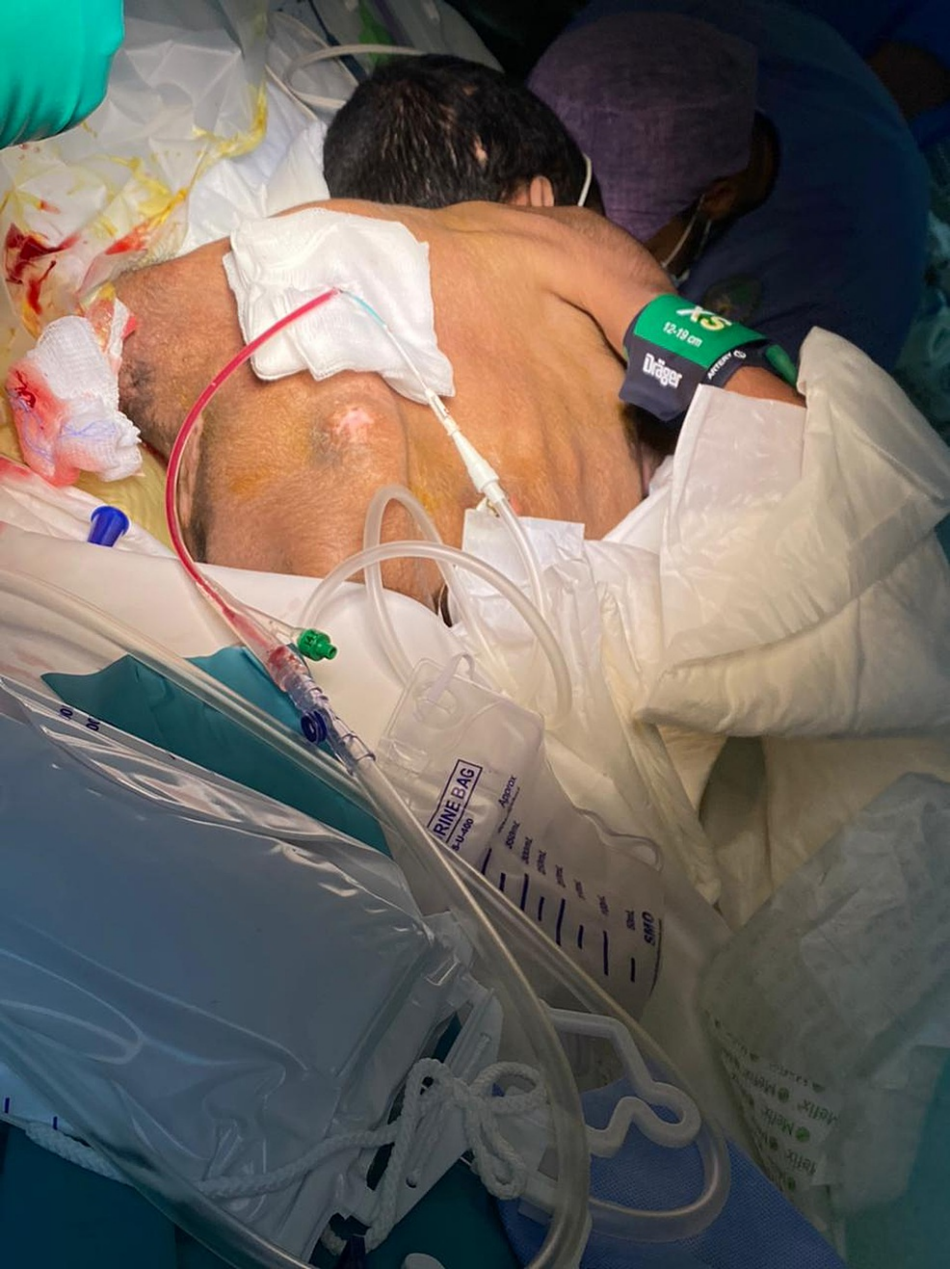
A postoperative picture of the patient after removing the stone and inserting the nephrostomy tube.

Balloon dilation was done up to 30 mmHg. A nephroscope was used, and the kidney stone was accessed and removed using an ultrasonic maneuver. After removing the stone, a nephrostomy tube was inserted. A proline stitch was applied, and the tube was fixed (Figure 5).

On the 2nd post-operative day, a CT scan was done and showed all tubes in place, and that the right kidney staghorn stone had completely resolved. The nephrostomy tube was removed, and the patient was discharged home in a good general condition without any complications. 

## Discussion

The abnormal curvature of the thoracic and/or lumbar regions of the vertebral column is responsible for spinal deformities such as scoliosis, kyphosis, and kyphoscoliosis. Abnormal body habitus in morbidly obese patients or in patients with spinal deformities makes urolithiasis treatment challenging for urologists as it creates technical difficulties that increase the risk of complication and reduces the likelihood of procedural success, thus deeming necessary innovative and inventive measures [8]. In patients with severe kyphosis, placement in the prone position is not feasible as it increases intraabdominal pressure and leads to secondary restriction of the diaphragm movement worsening respiratory restrictive function [9].

The European Association of Urology (EAU) recommends PCNL as the gold standard treatment for staghorn calculi, partial staghorn calculi, and stones larger than 20 mm [10]. The presence of a right staghorn renal stone was the indication of the PCNL procedure in our patient. Severe kyphoscoliosis, as in the case of our patient, can reduce the likelihood of achieving a high stone-free rate because it creates a challenging working space for the PCNL procedure. A two-step PCNL was opted for our patient. Thus, patient positioning was a crucial factor and is the main problem addressed in this study.

In 2010, eight patients with severe spinal deformities and renal calculi were successfully treated by Goumas-Kartalas and Montanari using ultrasound-guided PCNL [8]. Also, X-ray-guided PCNL was successfully employed by Kara et al., to treat five patients with scoliosis and renal calculi in 2011 [11]. These studies both indicated that PCNL is a viable option for patients with spinal deformities. Ultrasound guidance for determining the puncture pathway can minimize the risk of intraoperative damage to abdominal organs due to spinal deformity. Concurring with the aforementioned statement, our patient did not suffer from intraoperative internal organ damage. In comparison with the two-step PCNL group, the one-step PCNL group requires fewer analgesics and fares better in terms of surgery-related pain [8]. However, severe kyphoscoliosis deemed one-step PCNL to be non-suitable for our patient.

An important aspect to consider in kyphoscoliosis patients undergoing PCNL is pulmonary dysfunction. In kyphoscoliosis patients, the effective volume of the chest decreases. Consequently, this will results in a decrease of lung compliance, an increase in respiratory and circulatory resistance, and a dysfunction of blood-gas exchange in the pulmonary system [12]. Thus, in order to reduce fluid absorption and avoid volume overload, it is advised to use a low-pressure system [13]. Similar to our patient, general anesthesia should also be used along with correct positioning depending on the spinal curvature in order to avoid chest over-compression as much as possible. Epidural anesthesia should be avoided due to the burden it puts on the heart and lungs which will increase the operative risk in such patients [13].

## Conclusions

For kidney stone treatment in patients with spinal deformities, PCNL is not only a minimally invasive but also a safe and effective treatment option when done under correct precautions despite the challenges it poses on the surgeon and the interventional radiologist. According to the literature evidence, the success rate is high, and the morbidity rate is low. This case further adds to the evidence that ultrasound-guided percutaneous nephrolithotomy is a safe and effective approach in treating renal calculi in patients with kyphoscoliosis. The two most important parameters to consider are the chosen position depending on the angle of the spinal curvature and the type of anesthesia used. Also, an extensive evaluation of the patient's cardiopulmonary function is essential to ensure the safety of the procedure.
